# Supplementary material for: The CCR4–NOT Deadenylase Complex Maintains Adipocyte Identity
Source: Int J Mol Sci. 2019 Oct 24;20(21):5274. doi: 10.3390/ijms20215274 (PMC6862216; doi:10.3390/ijms20215274)
Supplement: Supplementary file 1 [file ijms-20-05274-s001.zip › Supplementary materials/Supplementary figure 1.pdf]

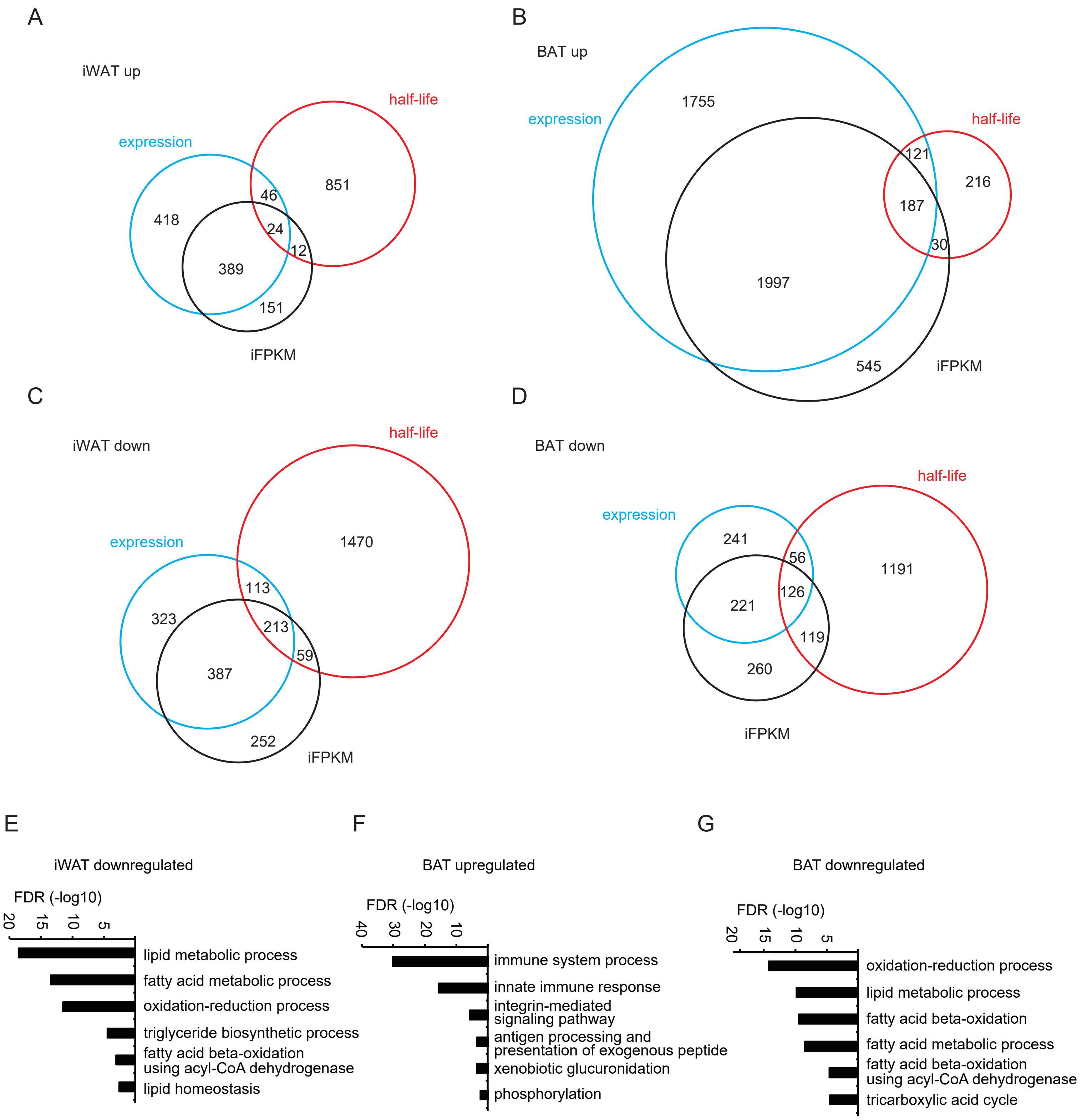

Supplementary figure 1

(A, B) Venn diagrams showing overlap of increased expression (blue), increased iFPKM (black) and elongated half-lives (red) in iWAT (A) or BAT (B) in *Cnot1*-AKO mice,

(C, D) Venn diagrams showing overlap of decreased expression (blue), decreased iFPKM (black) and shortened half-lives (red) in iWAT (C) or BAT (D) in *Cnot1*-AKO mice,

(E-G) GO enrichment analysis of genes common between decreased expression and iFPKM in iWAT (E) and BAT (G) of *Cnot1*-AKO mice and increased expression and iFPKM in BAT of *Cnot1*-AKO mice (F). Note that no significant enrichment of GO terms in genes common between increased expression and iFPKM in iWAT of *Cnot1*-AKO mice. Bar charts of the Biological Process GO terms (at most six) ranked by FDR (< 0.05) are shown.
