# Supplementary material for: The CCR4–NOT Deadenylase Complex Maintains Adipocyte Identity
Source: Int J Mol Sci. 2019 Oct 24;20(21):5274. doi: 10.3390/ijms20215274 (PMC6862216; doi:10.3390/ijms20215274)
Supplement: Supplementary file 1 [file ijms-20-05274-s001.zip › Supplementary materials/Supplementary Table 7 primer list.docx]

Supplementary Table 7. Primer sequences used for Real-time PCR in this study

| Gene | Forward primer | Reverse primer |
| --- | --- | --- |
| *Pparγ1* | 5’-TGAAAGAAGCGGTGAACCACTG-3’ | 5’-TGGCATCTCTGTGTCAACCATG-3’ |
| *Pparγ2* | 5’-GTTTTATGCTGTTATGGGTG-3’ | 5’-GTAATTTCTTGTGAAGTGCTCATAG-3’ |
| *Pck1* | 5’-ATCATCTTTGGTGGCCGTAG-3’ | 5’-CATGGCTGCTCCTACAAACA-3’ |
| *Myogenin* | 5’-CCTTGCTCAGCTCCCTCA-3’ | 5’-TGGGAGTTGCATTCACTGG-3’ |
| *Myod1* | 5’-AGCACTACAGTGGCGACTCA-3’ | 5’-GGCCGCTGTAATCCATCA-3’ |
| *Gapdh* | 5’-TGTCCGTCGTGGATCTGAC-3’ | 5’-CCTGCTTCACCACCTTCTTG-3’ |
