# Supplementary material for: The CCR4–NOT Deadenylase Complex Maintains Adipocyte Identity
Source: Int J Mol Sci. 2019 Oct 24;20(21):5274. doi: 10.3390/ijms20215274 (PMC6862216; doi:10.3390/ijms20215274)
Supplement: Supplementary file 1 [file ijms-20-05274-s001.zip › Supplementary materials/Supplementary Table legends.docx]

Supplementary table legends

**Supplementary table 1** GO analysis of differently expressing mRNAs in iWAT of *Cnot1*-AKO mice.

We analyzed enriched GO terms of mRNAs upregulated (upper) or downregulated (lower) more than 2-fold in iWAT of *Cnot1*-AKO mice compared to those of control mice using DAVID. P-values, false discovery rates (FDRs) and gene lists included in GO terms are summarized. Please see also Figure 4C.

**Supplementary table 2** GO analysis of differently expressing mRNAs in BAT of *Cnot1*-AKO mice.

We analyzed enriched GO terms of mRNAs upregulated more than 2.5-fold (upper) or downregulated more than 2-fold (lower) in BAT of *Cnot1*-AKO mice compared to those of control mice using DAVID. P-values, FDRs and gene lists included in GO terms are summarized. Please see also Figure 4D.

**Supplementary table 3** GO analysis of stabilized or destabilized mRNAs in mature adipocytes prepared from iWAT of *Cnot1*-AKO mice

We analyzed enriched GO terms of mRNAs that were stabilized (half-life *Cnot1*-AKO / control ratio >2.0, upper)) or destabilized (half-life *Cnot1*-AKO / control ratio <0.5, lower) in mature adipocytes prepared from iWAT of *Cnot1*-AKO mice than those of control mice using DAVID. P-values, FDRs and gene lists included in GO terms are summarized. Please see also Figure 5C.

**Supplementary table 4** GO analysis of destabilized mRNAs in mature adipocytes prepared from BAT of *Cnot1*-AKO mice

We analyzed enriched GO terms of mRNAs that were destabilized (half-life *Cnot1*-AKO / control ratio <0.5) in mature adipocytes prepared from BAT of *Cnot1*-AKO mice than those of control mice using DAVID. P-values, FDRs and gene lists included in GO terms are summarized. Please see also Figure 5D.

**Supplementary table 5** GO analysis of differently expressing pre-mRNAs in iWAT of *Cnot1*-AKO mice.

We analyzed enriched GO terms of genes showing intronic FPKMs that increased (upper) or decreased (lower) more than 2-fold in iWAT of 12-week-old *Cnot1*-AKO mice using DAVID. P-values, FDRs and gene lists included in GO terms are summarized. Please see also Figure 6C.

**Supplementary table 5** GO analysis of differently expressing pre-mRNAs in BAT of *Cnot1*-AKO mice.

We analyzed enriched GO terms of genes showing intronic FPKMs that increased (upper) or decreased (lower) more than 2-fold in BAT of 12-week-old *Cnot1*-AKO mice using DAVID. P-values, FDRs and gene lists included in GO terms are summarized. Please see also Figure 6D.

**Supplementary table 7**

Sequence lists of primers used in qPCR.
